# Supplementary material for: Social media research: We are publishing more but with weak influence
Source: PLoS One. 2024 Feb 8;19(2):e0297241. doi: 10.1371/journal.pone.0297241 (PMC10852228; doi:10.1371/journal.pone.0297241)
Supplement: Attachment — Submitted filename: Response to Reviewers.docx [file pone.0297241.s002.docx]

**We are very grateful to the editorial office for the comments and thoughtful suggestions. Based on these comments and suggestions, we have made careful modifications to the original manuscript.**

**In an effort to facilitate clarity and ease of identification, all the new changes and adjustments have been highlighted in blue in the revised manuscript.**

**Comments to the Author**

1. Is the manuscript technically sound, and do the data support the conclusions?

The manuscript must describe a technically sound piece of scientific research with data that supports the conclusions. Experiments must have been conducted rigorously, with appropriate controls, replication, and sample sizes. The conclusions must be drawn appropriately based on the data presented.

Reviewer #1: Partly

Reviewer #2: No

2. Has the statistical analysis been performed appropriately and rigorously?

Reviewer #1: N/A

Reviewer #2: No

3. Have the authors made all data underlying the findings in their manuscript fully available?

The [PLOS Data policy](https://ddec1-0-en-ctp.trendmicro.com/wis/clicktime/v1/query?url=http%3a%2f%2fwww.plosone.org%2fstatic%2fpolicies.action%23sharing&umid=afe227ef-aa63-42de-87ce-6af0bb6971a1&auth=8d3ccd473d52f326e51c0f75cb32c9541898e5d5-41c0ce16677868348b1d6636cc26b4cf971c003b) requires authors to make all data underlying the findings described in their manuscript fully available without restriction, with rare exception (please refer to the Data Availability Statement in the manuscript PDF file). The data should be provided as part of the manuscript or its supporting information, or deposited to a public repository. For example, in addition to summary statistics, the data points behind means, medians and variance measures should be available. If there are restrictions on publicly sharing data—e.g. participant privacy or use of data from a third party—those must be specified.

Reviewer #1: No

Reviewer #2: No

4. Is the manuscript presented in an intelligible fashion and written in standard English?

PLOS ONE does not copyedit accepted manuscripts, so the language in submitted articles must be clear, correct, and unambiguous. Any typographical or grammatical errors should be corrected at revision, so please note any specific errors here.

Reviewer #1: No

Reviewer #2: Yes

5. Review Comments to the Author

Please use the space provided to explain your answers to the questions above. You may also include additional comments for the author, including concerns about dual publication, research ethics, or publication ethics. (Please upload your review as an attachment if it exceeds 20,000 characters)

Reviewer #1: I am pleased to have a chance to review this manuscript. The manuscript discusses about social media research and its gap between the theory and practice. The title and the content of the paper is interesting and informative for the academic society. However, the structure and methodology of the paper is not aligned with health research methodology. A review paper in health research should obey the principles of standard checklists of reviews like PRISMA. The authors need to address few observations noted as below:

1- The reference style must be checked according to the journal recommendations. **We have completed the necessary adjustments to the reference style in accordance with the journal's recommendations, as per your feedback. We appreciate your diligence in pointing out this issue and providing guidance for improvement.**
2- Figures should have title in main manuscript. **We have made the necessary revisions to include titles for the figures in the main manuscript, as per your feedback.**
3- In the systematic review author did not mention the date of search and databases. If it was the same as bibliographic review, it should be mentioned. **We have addressed the issue regarding the omission of the search date, as per your feedback.**
4- The author should clarify the inclusion and exclusion criteria in systematic review. **Thanks for the suggestion. This is done in the revised manuscript**
5- In the first use of abbreviation, the full word must be written. **We have ensured that the full word is written out upon the initial use of each abbreviation.**
6- The author should use the past perfect tense when talking about actions related to conducting a study that occurred in the past and are completed. **Thanks for the comment. We made the necessary adjustments to ensure that the past perfect tense is correctly used when describing actions related to conducting the study that occurred in the past and are completed. This modification enhances the clarity and accuracy of the narrative.**
7- The first sentence of study 1 should better be changed to:” We conducted a bibliographic study,…” **Done**
8- In study 1, the authors wrote that “we first identified three keywords related to our study” but they mentioned two key words. If the authors used the exact words, it should be in ("). **Thanks. This was adjusted in our revised manuscript**
9- It is better to use “excluded” instead of “dismissed” in study one, paragraph 2. **Done.**
10- How was the sample size calculated in study 2 and 3? **This was explained in our revised manuscript.**
11- In last paragraph of study one, make the following changes” VOS is superior to multidimensional scaling for constructing bibliometric analyses and maps (Van Eck and Waltman, 2009), so we did not involve multidimensional scaling.” **Done**
12- Correct the number of figure21, in last paragraph of study one. **DONE**
13- Correct the number of figure 32, n study two, paragraph4. **DONE**
14- Change the first sentence of fifth paragraph of study to, to following sentence: “Participants mentioned that they use the previously mentioned tools to mainly gain more information about the latest trends in the fields of social media and technology.” **DONE**
15- Put table 3 and its title in right place. **DONE**
16- In academic writing it is not common to use “etc”.  **DONE**

**We appreciate the careful review of Reviewer 1 and the valuable feedback**

Reviewer #2: The manuscript entitled "Social Media Research: We Are Publishing More but with Weak Influence" aims to provide a bibliometric analysis that indicates the evolution of academic research on social media. The issue is quite interesting. Still, after reading the manuscript, I see room for improvements, which I will subsequently outline and hope that this review may help the authors as they continue to work on the paper:
• The introduction can and should be improved with paragraphs that better link the narrative from the relevance of the topic under analysis to the originality of this study. Make it clear what the originality of the study is.

**We sincerely appreciate your thoughtful review of the manuscript and your perceptive feedback regarding the introduction. Your comments about strengthening the linkage between the relevance of the topic and the originality of the study are particularly valuable. We have taken your feedback and have implemented the following changes to the introduction:**

**Enhanced Narrative Flow: The introduction now effectively connects the relevance of the topic under analysis to the originality of the study. This modification ensures that readers can easily follow the logical progression of ideas.**

**Explicitly Stating Originality: To address your concern about clarifying the study's originality, we have included a specific paragraph that explicitly outlines the unique contributions and original aspects of this research.**

• The authors said on page 8 of the file: “First, we examine the theoretical foundations of academic marketing research. Second, the research design and methodology of our three investigations are then described. Our first study involves a social media research bibliometric analysis with the goal of describing the evolution and development of academic social media research. Our second study gathers feedback and information from social media practitioners. Our third study lists suggestions for academic researchers.” Normally, I think this also depends on the field, but a good article could have only one investigation, but it should be very well done. However, I am not sure that the agglomeration of several investigations within the same study brings more value if they are not done rigorously. Thus, for instance, for bibliometric study, it is not very clear the search protocol. There are some details mentioned but not enough. Also, why the search in the database was only until March 2022? We are now in September 2023… See for instance some other bibliometric studies where the search protocol is very clearly presented:
¬ Alshater, M. M., Atayah, F. O., & Khan, A. (2021). What do we know about business and economics research during COVID-19: A bibliometric review. Economic Research-EkonomskaIstra_zivanja, 1–29. https://ddec1-0-en-ctp.trendmicro.com:443/wis/clicktime/v1/query?url=https%3a%2f%2fdoi.org%2f10.1080%2f1331677X.2021.1927786&umid=afe227ef-aa63-42de-87ce-6af0bb6971a1&auth=8d3ccd473d52f326e51c0f75cb32c9541898e5d5-117b12e2d0d425e348c4696cbc40cffb2df56e72
¬ Bota-Avram, C (2023). Bibliometric analysis of sustainable business performance: where are we going? A science map of the field. Economic Research-Ekonomska Istraživanja, 36 (1), 2137-2176. https://ddec1-0-en-ctp.trendmicro.com:443/wis/clicktime/v1/query?url=https%3a%2f%2fdoi.org%2f10.1080%2f1331677X.2022.2096094&umid=afe227ef-aa63-42de-87ce-6af0bb6971a1&auth=8d3ccd473d52f326e51c0f75cb32c9541898e5d5-f2951e34501c62cc140391b75cdc6d9df37ca3d3
¬ Caputo, A., Pizzi, S., Pellegrini, M., & Dabic, M. (2021). Digitalization and business models: Where are we going? A science map of the field. Journal of Business Research, 123, 489–501. https://ddec1-0-en-ctp.trendmicro.com:443/wis/clicktime/v1/query?url=https%3a%2f%2fdoi.org%2f10.1016%2fj.jbusres.2020.09.053&umid=afe227ef-aa63-42de-87ce-6af0bb6971a1&auth=8d3ccd473d52f326e51c0f75cb32c9541898e5d5-6069368ff80ce43a31060c99ad694f05ab232ef3
¬ Wang, X., Xu, Z., Su, S.-F., & Zhou, W. (2021). A comprehensive bibliometric analysis of uncertain group decision making from 1980 to 2019. Information Sciences, 547, 328–353. https://ddec1-0-en-ctp.trendmicro.com:443/wis/clicktime/v1/query?url=https%3a%2f%2fdoi.org%2f10.1016%2fj.ins.2020.08.036&umid=afe227ef-aa63-42de-87ce-6af0bb6971a1&auth=8d3ccd473d52f326e51c0f75cb32c9541898e5d5-a62934572fd1feaa6e777452bf434a925b841820
¬ Wang, X., Xu, Z., Qin, Y., & Skare, M. (2021). Service networks for sustainable business: A dynamic evolution analysis over half a century. Journal of Business Research, 136, 543–557. https://ddec1-0-en-ctp.trendmicro.com:443/wis/clicktime/v1/query?url=https%3a%2f%2fdoi.org%2f10.1016%2fj.jbusres.2021.07.062&umid=afe227ef-aa63-42de-87ce-6af0bb6971a1&auth=8d3ccd473d52f326e51c0f75cb32c9541898e5d5-422e2488d0ec9111726c1821d7208ad524e01973
**Thanks for the suggestions and the references. We took this comment into consideration and re-structured our bibliometric study accordingly.**
• The bibliometric study is very superficial made, normally a good bibliometric study supposes to present some performance analysis and also some science mapping indicators. See for instance a very valuable article with a useful guide on how to conduct a bibliometric study. Otherwise, because the authors selected articles and analysed only the co-occurrence of the keywords from there, it is far from a valuable bibliometric analysis. Also, the authors must explain what bibliometric indicators were analysed and why?!
¬ Donthu, N., Kumar, S., Mukherjee, D., Pandey, N., & Lim, W. M. (2021). How to conduct a bibliometric analysis: An overview and guidelines. Journal of Business Research, 133, 285–296. https://ddec1-0-en-ctp.trendmicro.com:443/wis/clicktime/v1/query?url=https%3a%2f%2fdoi.org%2f10.1016%2fj.jbusres.2021.04.070&umid=afe227ef-aa63-42de-87ce-6af0bb6971a1&auth=8d3ccd473d52f326e51c0f75cb32c9541898e5d5-29fb859f29c949fa50f2979d6d228cf1d6cfff0a

**Thank you for your constructive feedback. In response to your observation that the bibliometric study appears superficial, we have expanded our analysis to include performance metrics and science mapping indicators. This addition significantly enriches the study by providing a more comprehensive view.**
• Then, at page 12. The authors said: “In our systematic literature review…” but hold on….it is a bibliometric study or a systematic literature review? My impression is that the authors don’t know the difference between them, they think it is the same thing but definitely they are not the same. See also the article where the difference between them is explained.
• Donthu, N., Kumar, S., Mukherjee, D., Pandey, N., & Lim, W. M. (2021). How to conduct a bibliometric analysis: An overview and guidelines. Journal of Business Research, 133, 285–296. https://ddec1-0-en-ctp.trendmicro.com:443/wis/clicktime/v1/query?url=https%3a%2f%2fdoi.org%2f10.1016%2fj.jbusres.2021.04.070&umid=afe227ef-aa63-42de-87ce-6af0bb6971a1&auth=8d3ccd473d52f326e51c0f75cb32c9541898e5d5-29fb859f29c949fa50f2979d6d228cf1d6cfff0a
• However, this first study does look at all as a systematic lit review. A systematic literature review is supposed to follow a protocol (see, for instance, the PRISMA protocol)
• Also, what is the utility of bibliometric analysis if no future research directions are not identified based on bibliometric analysis??!!

**Your comments have prompted us to revisit and refine our approach to better align with best practices and provide more actionable insights. You are absolutely right that a systematic literature review should adhere to a protocol for rigorous and transparent selection and evaluation of studies. We have revised our manuscript to explicitly state that our study is not a systematic literature review. Instead, we have categorized our review as a comprehensive bibliographic analysis. This reclassification accurately reflects our methodology and approach**
• Regarding the study no.2, the authors adopt the same superficial approach. No literature review as background for study 2 is indicated. Again, it is not clear how the database with respondents was selected. How the database with 280 social media practitioners was selected? Based on what criteria? Much more rigour would be needed in an academic paper…
**We appreciate your comments about the need for a more rigorous approach and greater clarity in presenting the background and methodology. We have taken your input and have made the following improvements: We understand the importance of providing a strong foundation for our second study. To address this concern, we have included a background, which sets the context for the study and underscores its significance within the existing body of research. This addition ensures that readers have the necessary background to understand the study's objectives and contributions.**

**We also acknowledge your concern about the selection criteria for the database of 280 social media practitioners. In response, we have included a dedicated section in the manuscript that outlines the specific criteria used for selecting respondents. This includes details about the professional qualifications, experience, and other relevant characteristics that guided our participant selection process**
• In my opinion, this article is a compilation of insufficiently and non-rigorously developed studies. As a scholarship reader, I would prefer to see a single study with the necessary academic rigour rather than an agglomeration of studies insufficiently developed within the same article.

**We understand your preference for a single study with comprehensive academic rigor, and we agree that cohesion and integration among the studies are essential for a cohesive article. In response to your observations, we have taken the following actions to enhance the structure and flow, we have revised the article to create a stronger sense of cohesion and integration among the three studies. This involves improving the transitions between studies and clearly articulating how each study complements and builds upon the other**
• As a value-added bibliometric analysis, I was expected to see at least a brief discussion on the most popular topics ( a content analysis), from where a useful summary of future research directions could be developed for the researchers interested in this field. In addition, a discussion of the emerging trends in research for the field under investigation would be very useful for the readers.

**In response to your suggestion, we have included a dedicated section in the article that discusses emerging trends in research within the field. This discussion identifies and elaborates on the latest developments and areas of interest, helping readers stay current with the evolving landscape of social media.**
FINAL COMMENT
I know the effort it takes to develop rigorous, serious, and useful research work. The comments I have just made are intended to help you better your scientific work and reflect only my point of view. I hope these comments and suggestions can help improve this paper.
Good work and good luck with this journey!

**Thank you very much for your understanding and kind words. We genuinely appreciate your time and effort in providing constructive feedback and suggestions to improve our research work. Your perspective and expertise have been invaluable in refining our manuscript, and your support is deeply appreciated.**

6. PLOS authors have the option to publish the peer review history of their article ([what does this mean?](https://ddec1-0-en-ctp.trendmicro.com/wis/clicktime/v1/query?url=https%3a%2f%2fjournals.plos.org%2fplosone%2fs%2feditorial%2dand%2dpeer%2dreview%2dprocess%23loc%2dpeer%2dreview%2dhistory&umid=afe227ef-aa63-42de-87ce-6af0bb6971a1&auth=8d3ccd473d52f326e51c0f75cb32c9541898e5d5-48f9f74f211b0f94c2f0e891e2b86667d1c1cc53)). If published, this will include your full peer review and any attached files.


If you choose “no”, your identity will remain anonymous but your review may still be made public.
